# Hypothermia Induced by Oxcarbazepine after Transient Forebrain Ischemia Exerts Therapeutic Neuroprotection through Transient Receptor Potential Vanilloid Type 1 and 4 in Gerbils

**DOI:** 10.3390/ijms23010237

**Published:** 2021-12-27

**Authors:** Hyung-Il Kim, Jae-Chul Lee, Dae Won Kim, Myoung Cheol Shin, Jun Hwi Cho, Ji Hyeon Ahn, Soon-Sung Lim, Il Jun Kang, Joon Ha Park, Moo-Ho Won, Tae-Kyeong Lee

**Affiliations:** 1 Department of Emergency Medicine, Dankook University Hospital, College of Medicine, Dankook University, Cheonan 31116, Chungnam, Korea; hilovesjj@naver.com; 2 Department of Emergency Medicine, Kangwon National University Hospital, School of Medicine, Kangwon National University, Chuncheon 24289, Gangwon, Korea; dr10126@naver.com (M.C.S.); cjhemd@kangwon.ac.kr (J.H.C.); 3 Department of Neurobiology, School of Medicine, Kangwon National University, Chuncheon 24341, Gangwon, Korea; anajclee@kangwon.ac.kr (J.-C.L.); jh-ahn@ysu.ac.kr (J.H.A.); 4 Department of Biochemistry and Molecular Biology, Research Institute of Oral Sciences, College of Dentistry, Gangnung-Wonju National University, Gangneung 25457, Gangwon, Korea; kimdw@gwnu.ac.kr; 5 Department of Physical Therapy, College of Health Science, Youngsan University, Yangsan 50510, Gyeongnam, Korea; 6 Department of Food Science and Nutrition, Hallym University, Chuncheon 24252, Gangwon, Korea; limss@hallym.ac.kr (S.-S.L.); ijkang@hallym.ac.kr (I.J.K.); 7 Department of Anatomy, College of Korean Medicine, Dongguk University, Gyeongju 38066, Gyeongbuk, Korea; jh-park@dongguk.ac.kr

**Keywords:** antiepileptic drug, cognition, hippocampus, ischemia–reperfusion, pyramidal cells, transient receptor potential cation channels

## Abstract

In the present study, we investigated the neuroprotective effect of post-ischemic treatment with oxcarbazepine (OXC; an anticonvulsant compound) against ischemic injury induced by transient forebrain ischemia and its mechanisms in gerbils. Transient ischemia was induced in the forebrain by occlusion of both common carotid arteries for 5 min under normothermic conditions (37 ± 0.2 °C). The ischemic gerbils were treated with vehicle, hypothermia (whole-body cooling; 33.0 ± 0.2 °C), or 200 mg/kg OXC. Post-ischemic treatments with vehicle and hypothermia failed to attenuate and improve, respectively, ischemia-induced hyperactivity and cognitive impairment (decline in spatial and short-term memory). However, post-ischemic treatment with OXC significantly attenuated the hyperactivity and the cognitive impairment, showing that OXC treatment significantly reduced body temperature (to about 33 °C). When the hippocampus was histopathologically examined, pyramidal cells (principal neurons) were dead (lost) in the subfield Cornu Ammonis 1 (CA1) of the gerbils treated with vehicle and hypothermia on Day 4 after ischemia, but these cells were saved in the gerbils treated with OXC. In the gerbils treated with OXC after ischemia, the expression of transient receptor potential vanilloid type 1 (TRPV1; one of the transient receptor potential cation channels) was significantly increased in the CA1 region compared with that in the gerbils treated with vehicle and hypothermia. In brief, our results showed that OXC-induced hypothermia after transient forebrain ischemia effectively protected against ischemia–reperfusion injury through an increase in TRPV1 expression in the gerbil hippocampal CA1 region, indicating that TRPV1 is involved in OXC-induced hypothermia.

## 1. Introduction

Body temperature can affect the results of brain ischemia [1,2,3]. Hypothermia is one of the available therapies for stroke, as evaluated by the Stroke Therapy Academic Industry Roundtable criteria [4]. Neuroprotective effects by hypothermia have been established in experimental models [5,6]. While the exact mechanism by which hypothermia protects against neuronal cell death is unknown, hypothermia is likely to act on multiple pathways to ultimately prevent neuronal cell death [7,8]. In most clinical studies, hypothermia is caused by body surface cooling. Unfortunately, this method of forced cooling is slow and cumbersome. It takes several hours to reach target core body temperature, and it should be closely monitored to ensure that the target temperature is achieved [9]. Recently, methods using agents for stroke therapy have been suggested as a way to reduce core body temperature more efficiently and quickly than surface cooling [10,11,12]. Thus, the uses of agent-induced hypothermia may help overcome the above-mentioned obstacles. 

Many studies have shown that antiepileptic drugs have beneficial effects to counteract neuronal damage from experimentally induced brain damage, such as ischemic stroke, intracerebral hemorrhage, and traumatic brain injury [13,14,15]. OXC is an anticonvulsant compound commonly used in epilepsy treatment [16]. The great mechanism of OXC is reported to inhibit voltage-dependent sodium channels [17]. In addition, OXC exerts beneficial effects through preventing the release of extracellular glutamate and changing recurrent depolarization [18]. Furthermore, OXC reduces Ca^2+^ influx through blockade of the synaptic Ca^2+^ channels in rat hippocampal slices [19]. In particular, we recently reported that OXC treatment after transient forebrain ischemia in gerbils conferred neuroprotection against ischemic injury by activating the Nrf2 defense pathway [20]. However, the mechanism of OXC’s neuroprotective effect against ischemic damage remains unclear. 

It has been reported that transient receptor potential cation channels (TRP channels) are pivotal in temperature regulation [21,22]. Among the members of the TRP channels, transient receptor potential vanilloid type 1 (TRPV1), which is also called capsaicin–vanilloid receptor-1, is widely expressed in the central nervous system (CNS) [23,24] and plays a significant role in thermoregulation [25,26,27]. In addition, TRPV4, as a non-selective cation channel, responds to mechanical, thermal, and chemical stimuli [28,29]. Despite evidence suggesting that TRPVs have diverse physiological roles in the CNS, little is known about their pathophysiological roles in neurological disorders, especially in cerebral ischemic injury. Based on the functions of TRPVs, therefore, the objective of this study was to investigate therapeutic effects of OXC against ischemic injury and its mechanisms in the hippocampus, which is very vulnerable to ischemia–reperfusion injury, after transient forebrain ischemia (tFI) in gerbils. In addition, the therapeutic effects of OXC against ischemic injury were compared with those by whole-body cooling (hypothermia). 

## 2. Results

### 2.1. Body Temperature

**Figure 1:**
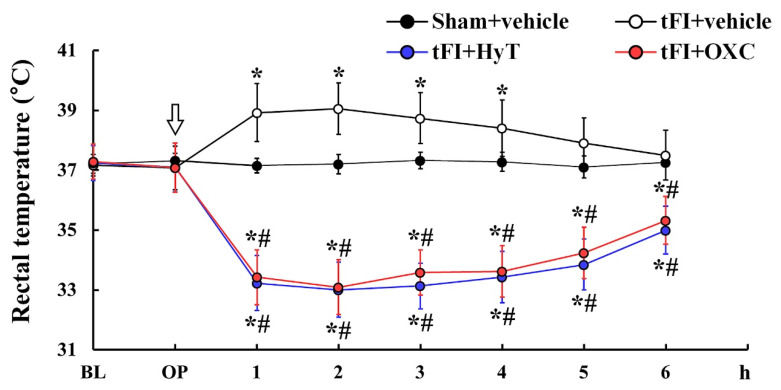
Changes in body temperature for six hours after tFI or sham operation. Body temperature was significantly low in the tFI+HyT and tFI+OXC groups when compared with that in the tFI+vehicle group. The white arrow indicates the time of hypothermia induction and OXC treatment. The bars indicate the means ± SEM (*n* = 7 for each group; * p *<* 0.05 vs. sham+vehicle group, # *p* < 0.05 vs. corresponding time of tFI+vehicle group). BL, base line; OP, operation (sham or to induce tFI).

As shown in Figure 1, the body temperature of gerbils treated with vehicle (saline) after a sham operation (sham+vehicle group) was not altered after the operation. However, in gerbils treated with vehicle after tFI (tFI+vehicle group), body temperature was suddenly elevated (to about 39 °C) at one and two hours after tFI and thereafter gradually decreased (normothermia at six hours after tFI). In gerbils treated with OXC after tFI (tFI+OXC group), body temperature was significantly decreased (to about 33 °C) at one and two hours after tFI and thereafter gradually increased (to about 35 °C at six hours after tFI): the effect was statistically significant (*p* < 0.05) when compared with the tFI+vehicle group (Figure 1). In gerbils treated with hypothermia (HyT, whole-body cooling) (tFI+HyT group), the change in body temperature was similar to that shown in the tFI+OXC group.

### 2.2. Hyperactivity

**Figure 2:**
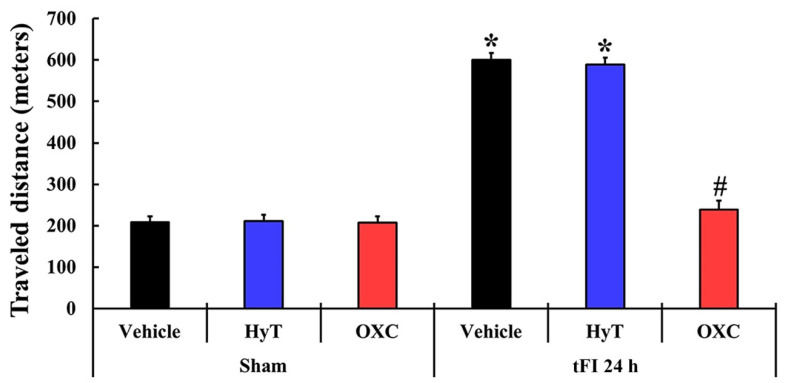
SMA test at 24 h after sham or tFI operation. In the tFI+vehicle and tFI+HyT groups, the mean traveled distances were significantly longer than that evaluated in the sham+vehicle group. In contrast, in the tFI+OXC group, the mean traveled distance was similar to that shown in the sham+vehicle group. The bars indicate the means ± SEM (*n* = 7 for each group; * *p* < 0.05 vs. sham+vehicle group and # *p* < 0.05 vs. corresponding time of the tFI+vehicle group).

To examine change in hyperactivity, the spontaneous motor activity (SMA) test was performed in all groups (Figure 2). In the sham+vehicle group, the traveled distance was 208.4 m on average. The mean traveled distances (211.0 m and 207.9 m, respectively) in the sham+HyT and sham+OXC groups were similar to that shown in the sham+vehicle group. However, in the tFI+vehicle and tFI+HyT groups, the mean traveled distances (600.1 m and 588.4 m, respectively) were significantly increased when compared with that shown in the sham+vehicle group. In contrast, the gerbils of the tFI+OXC group traveled a significantly shorter distance (239.1) as compared with those in the tFI+vehicle group.

### 2.3. Cognitive Functions

#### 2.3.1. Spatial Memory

**Figure 3:**
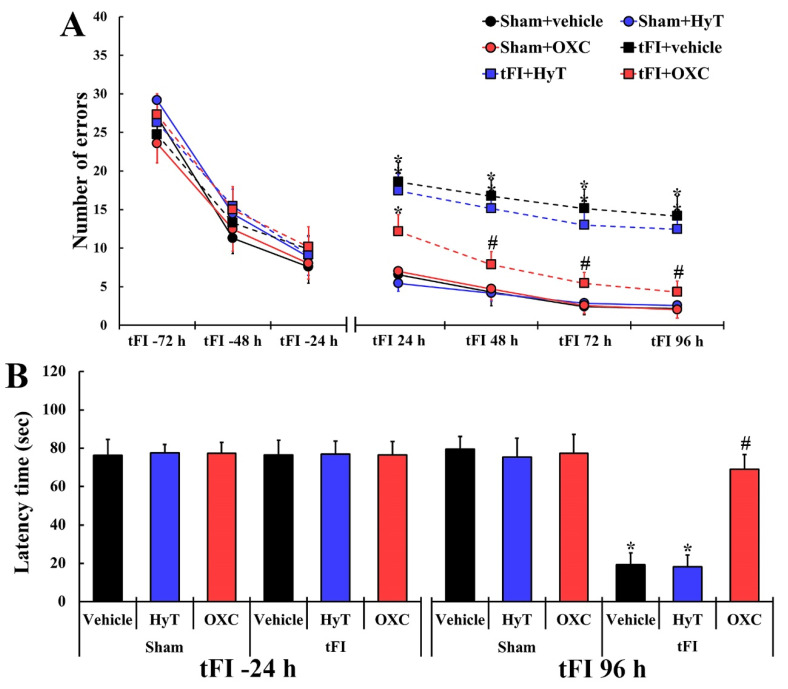
(**A**) The mean numbers of errors in the RAMT in all six groups at 72, 48, and 24 h before tFI/sham operation and 24, 48, 72, and 96 h after tFI/sham operation. The error number in the tFI+OXC group was significantly decreased from 48 h after tFI when compared with that in tFI+vehicle. (**B**) Latency time (seconds) in the PAT in all groups at 24 h before tFI/sham operation and 96 h after tFI/sham operation. The latency time shown in the tFI+OXC group was significantly increased when compared with that in the tFI+vehicle group. The bars indicate the means ± SEM (*n* = 7 for each group; * *p* < 0.05 vs. sham+vehicle group; # *p* < 0.05 vs. corresponding time of the tFI+vehicle group).

To examine change in spatial memory, the radial arm maze test (RAMT) was performed in all groups (Figure 3A). At one, two, and three days before the tFI or sham operation, changes in the numbers of errors were not significantly different across all groups: this finding indicates that all animals had undergone identical pre-training for RAMT. In all sham groups, a significant difference in the numbers of errors was not found every time (one day, two days, three days, and four days) after sham operation. On the other hand, the numbers of errors in the tFI+vehicle group were significantly higher than those shown in the sham+vehicle group on Days 1, 2, 3 and 4 after tFI operation. In addition, the numbers evaluated in the tFI+HyT group were similar to those in the tFI+vehicle group. However, in the tFI+OXC group, the numbers of errors were significantly lower than those shown in the tFI+vehicle group.

#### 2.3.2. Short-Term Memory 

The passive avoidance test (PAT) was performed to examine changes in short-term memory in all groups (Figure 3B). At one day before tFI or sham operation, significant differences in latency time were not detected across all groups: this finding implies that all gerbils had been subjected to an identical pre-training. In all sham groups, the latency time was evaluated on Day 4 after tFI and was similar to that found at one day before tFI. However, in the tFI+vehicle group, significantly shortened latency time was obtained on Day 1 after tFI. In addition, the latency time in the tFI+HyT group was similar to that in the tFI+vehicle group. In contrast, the latency time evaluated in the tFI+OXC group was significantly lengthened as compared to that shown in the tFI+vehicle group.

### 2.4. Levels of TRPV1 and TRPV4

**Figure 4:**
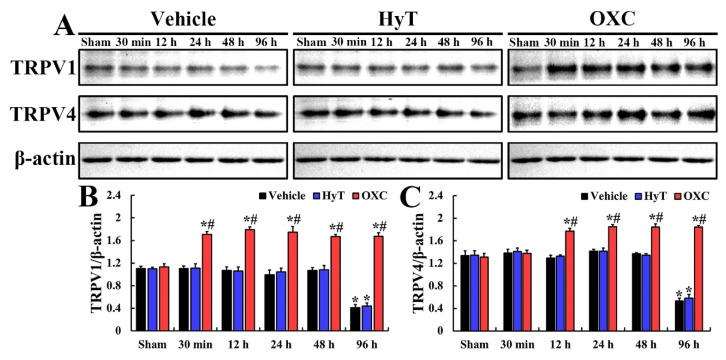
(**A**) Representative immunoblots of TRPV 1 in all groups at 30 min, 12, 24, 48, and 96 h after tFI or sham operation. In the tFI+vehicle and tFI+HyT groups, both TRPV1 and TRPV4 levels were not significantly altered until 48 h after tFI, but they were significantly lowered at 96 h after tFI as compared with the corresponding sham+vehicle group. In the tFI+OXC group, TRPV1 and TRPV4 levels were significantly enhanced at 30 min and 12 h, respectively, after tFI, and the increased levels were maintained until 96 h after tFI. (**B**,**C**) Protein levels normalized to β-actin of TRPV1 (**B**) and TRPV4 (**C**). The bars indicate the means ± SEM (*n* = 5 for each group; * *p* < 0.05 vs. sham+vehicle group, # *p* < 0.05 vs. corresponding time of the tFI+vehicle group).

Changes in the expression levels of TRPV1 and TRPV4 in the CA1 regions of all groups were examined using the Western blot technique (Figure 4). In all sham groups, the TRPV1 level was fundamentally detected, and no significant difference in TRPV1 level was found across the sham groups (Figure 4A,B). In the tFI+vehicle group, the TRPV1 level was maintained until two days after tFI (Figure 4A,B). Also, in the tFI+HyT group, the TRPV1 level was not significantly altered until two days after tFI (Figure 4A,B). In these two groups, at four days after tFI, a significant decrease in TRPV1 level was observed (37.1% and 39.8% vs. sham+vehicle group, respectively) (Figure 4A,B). However, in the tFI+OXC group, the TRPV1 level was significantly enhanced (154.8% vs. sham+vehicle group) at 30 min after tFI, and the increased TRPV1 level was maintained until four days after tFI (Figure 4A,B).

The TRPV4 level was fundamentally found in all sham groups, and no significant difference in the level was found across the sham groups (Figure 4A,C). In the tFI+vehicle and tFI+HyT groups, the TRPV4 level was not changed until two days after tFI (Figure 4A,C). In these groups, the TRPV4 level was significantly reduced (39.8% and 43.6% vs. sham+vehicle group, respectively) at four days after tFI (Figure 4A,C). In the tFI+OXC group, however, the TRPV4 level was significantly increased (132.1% vs. sham+vehicle group) at 12 h after tFI, and the enhanced level was maintained until four days after tFI (Figure 4A,C). 

### 2.5. TRPV1 and TRPV4 Immunoreactivity

**Figure 5:**
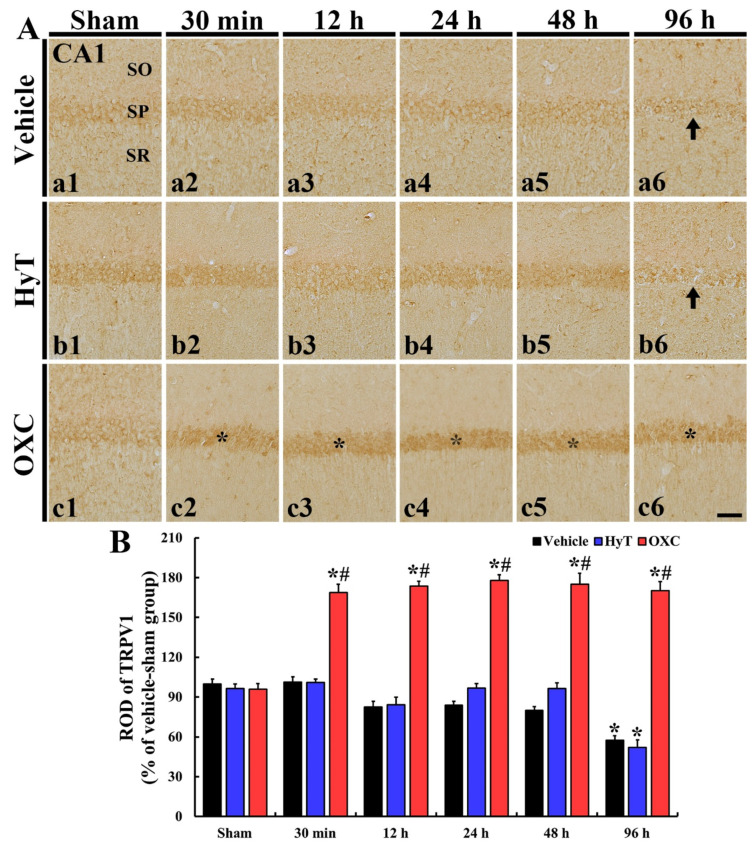
(**A**) Representative images of TRPV 1 immunohistochemistry in the CA1 region of all groups at 30 min, 12 h, 24 h, 48 h, and 96 h after tFI or sham operation. In the tFI+vehicle and tFI+HyT groups, TRPV1 immunoreactivity was not significantly changed in the stratum pyramidale (SP) until 48 h after tFI, but it was markedly reduced (arrows) at 96 h after tFI. In the tFI+OXC group, however, TRPV1 immunoreactivity was significantly increased in the SP at 30 min and maintained (asterisks) until 96 h after tFI. (**B**) Relative optical density (ROD) of TRPV1 immunoreactivity. The bars indicate the means ± SEM (*n* = 7 for each group; * *p* < 0.05 vs. sham+vehicle group, # *p* < 0.05 vs. corresponding time of the tFI+vehicle group). SO, stratum oriens; SR, stratum radiatum. Scale bars = 100 μm.

**Figure 6:**
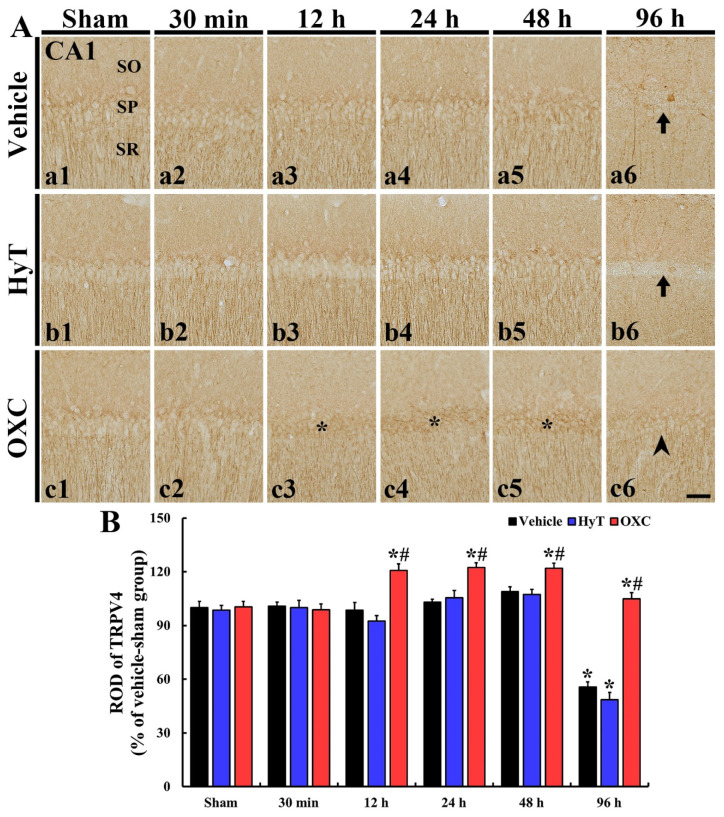
(**A**) Representative images of TRPV4 immunohistochemistry in the CA1 region of all groups at 30 min, 12 h, 1 day, 2 days, and 4 days after tFI or sham operation. In the tFI+vehicle and tFI+HyT groups, TRPV4 immunoreactivity was similar to that in the sham+vehicle group until 2 days after tFI, but it was dramatically reduced in the SP (arrows) at four days after tFI. In contrast, TRPV4 immunoreactivity of the tFI+OXC group was significantly increased (asterisks) in the SP at 12 h after tFI and slightly decreased (arrowhead) at four days after tFI. (**B**) ROD of TRPV4 immunoreactivity. The bars indicate the means ± SEM (*n* = 7 for each group; * *p* < 0.05 vs. sham+vehicle group, # *p* < 0.05 vs. corresponding time of the tFI+vehicle group). SO, stratum oriens; SR, stratum radiatum. Scale bars = 100 μm.

Changes in TRPV1 and TRPV4 immunoreactivity in the CA1 regions of all groups were investigated via immunohistochemistry (Figure 5 and Figure 6).

#### 2.5.1. TRPV1 Immunoreactivity

In all sham groups, TRPV1 immunoreactivity was fundamentally detected in the CA1 region: the immunoreactivity was mainly shown in the stratum pyramidale (SP), which consists of pyramidal cells (principal cells) (Figure 5A(a1–c1)).

In the tFI+vehicle group, TRPV1 immunoreactivity was not altered until two days after tFI as compared to the sham+vehicle group (Figure 5A(a2–a5),B). Also, in the tFI+HyT group, TRPV1 immunoreactivity was not significantly altered until two days after tFI (Figure 5A(b2–b5),B). In these two groups, however, TRPV1 immunoreactivity was markedly reduced in the SP (57.4% and 52.2% vs. sham+vehicle group, respectively) (Figure 5A(a6,b6),B). In the tFI+OXC group, TRPV1 immunoreactivity was significantly increased in the SP (168.9% vs. sham+vehicle group) at 30 min after tFI (Figure 5A(c2),B), and the increased TRPV1 immunoreactivity in the SP was maintained until four days after tFI (Figure 5A(c3–c6),B).

#### 2.5.2. TRPV4 Immunoreactivity

In all sham groups, TRPV4 immunoreactivity was fundamentally shown in the CA1 region, and the immunoreactivity was mainly expressed at the cytoplasmic periphery and dendrites of pyramidal cells located in the SP (Figure 6A(a1,b1,c1)).

In the tFI+vehicle and tFI+HyT groups, TRPV4 immunoreactivity observed until 48 h after tFI was similar to that shown in the sham groups (Figure 6A(a2–a5,b2–b5),B). In these two groups, significantly reduced TRPV4 immunoreactivity was detected in the somata and dendrites of the pyramidal cells (55.6% and 48.5% vs. sham+vehicle group, respectively) at 96 h after tFI (Figure 6A(a6,b6),B). In contrast, the TRPV4 immunoreactivity of the tFI+OXC group was significantly strengthened (168.9% vs. sham+vehicle group) at 12 h after tFI (Figure 6A(c3),B), was maintained until 48 h after tFI (Figure 6A(c4,c5),B), and became similar (104.9% vs. sham+vehicle group) to that in the sham+vehicle group at 96 h after tFI (Figure 6A(c6),B).

### 2.6. Neuroprotection

#### 2.6.1. Findings by Cresyl Violet (CV) Histochemistry

**Figure 7:**
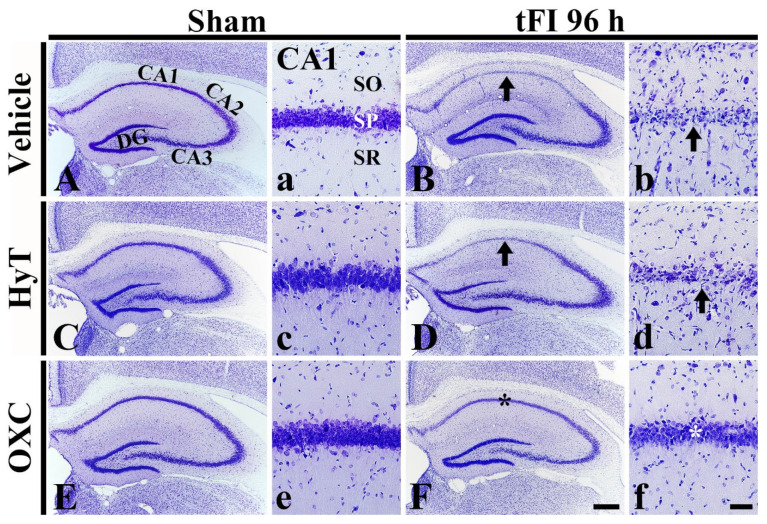
CV staining in the hippocampus and its CA1 region of the sham+vehicle (**A**,**a**), sham+HyT (**C**,**c**), sham+OXC (**E**,**e**), tFI+vehicle (**B**,**b**), tFI+HyT (**D**,**d**), and tFI+OXC (**F**,**f**) groups. In the tFI+vehicle and tFI+HyT groups, CV stainability was apparently reduced in the SP (arrows) of the CA1 region. In the tFI+OXC group, however, the morphology of CV^+^ cells was similar to that in the sham+vehicle group. The bars indicate the means ± SEM (*n* = 7 for each group). DG, dentate gyrus. Scale bars = 400 μm (**A**–**F**) and 100 μm (**a**–**f**).

We examined changes in cells located in the hippocampus after tFI or sham operation and whether OXC was associated with cell survival in the ischemic hippocampus using CV histochemical staining (Figure 7). In all sham groups, CV-stained (CV^+^) cells were easily identified in the hippocampus, and no difference in cellular distribution was shown across the sham groups: in particular, CV^+^ cells in the SP, which are called pyramidal cells and principal neurons, were relatively large and pyramid-like in shape (Figure 7A,C,E,a,c,e). In the tFI+vehicle group, CV stainability was apparently decreased in the SP of the CA1 region, but not in the CA2/3 region, at four days after tFI as compared with the sham+vehicle group (Figure 7B): at this point in time, CV^+^ pyramidal cells were apparently shrunken and had darkly condensed nuclei (Figure 4B). In the tFI+HyT group, the change in CV^+^ cells at four days after tFI was similar to that in the tFI+vehicle group (Figure 7D,d). However, in the tFI+OXC group, CV^+^ cells at four days after tFI were not altered when compared to those in the tFI+vehicle group (Figure 7F,f). This finding means that post-ischemic OXC treatment saved CA1 pyramidal cells from ischemic injury.

#### 2.6.2. Findings by Neuronal Nuclei (NeuN) Immunohistochemistry and Fluoro-Jade B (F-J B) Staining

**Figure 8:**
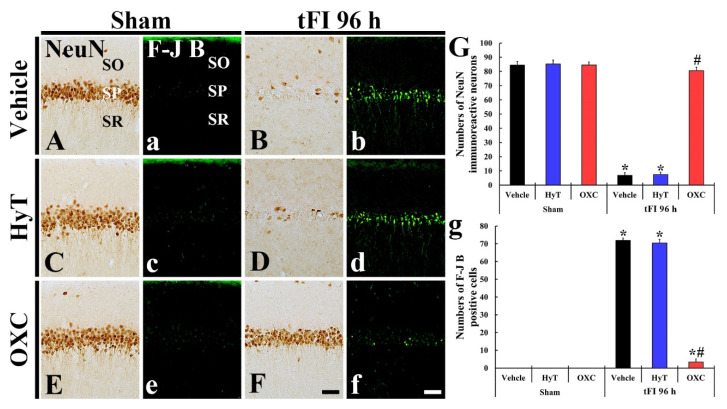
(**A**–**F** and **a**–**f**) NeuN immunohistochemistry (**A**–**F**) and F-J B staining (**a**–**f**) in the CA1 region of the sham+vehicle (**A**,**a**), sham+HyT (**C**,**c**), sham+OXC (**E**,**e**), tFI+vehicle (**B**,**b**), tFI+HyT (**D**,**d**), and tFI+OXC (**F**,**f**) groups on Day 4 after tFI or sham operation. Few NeuN^+^ and many F-J B^+^ cells were observed in the SP of the tFI+vehicle and tFI+HyT groups. However, in the tFI+OXC group, many NeuN^+^ and few F-J B^+^ cells were found in the SP on Day 4 after tFI. (**G**,**g**) Mean numbers of NeuN^+^ (**G**) and F-J B^+^ (**g**) cells. The bars indicate the means ± SEM (*n* = 7 for each group; * *p* < 0.05 vs. sham+vehicle group, # *p* < 0.05 vs. corresponding time of the tFI+vehicle group). SO, stratum oriens; SR, stratum radiatum. Scale bars = 100 μm.

We examined neuronal change and death (loss) in the CA1 region using NeuN (a marker for neurons) immunohistochemistry and F-J B (a fluorescent marker for neurodegeneration) staining, respectively (Figure 8). In all sham groups, intact pyramidal cells (neurons) (84.4, 85.3, and 84.6 cells/250 μm^2^ in the sham+vehicle, sham+HyT, and sham+OXC group, respectively) located in the SP were strongly immunostained with NeuN (Figure 8A,C,E,G). In these sham groups, F-J B^+^ cells were not detected in the CA1 region (Figure 8a,c,e,g). However, in the tFI+vehicle group, few NeuN^+^ cells (6.9 cells/250 μm^2^) and many F-J B^+^ cells (71.9 cells/250 μm^2^) were observed in the SP on Day 4 after tFI (Figure 8B,b,G,g). Also, few NeuN^+^ and many F-J B^+^ cells (7.4 cells/250 μm^2^ and 70.4 cells/250 μm^2^, respectively) were observed in the tFI+HyT group on Day 4 after tFI (Figure 8D,d,G,g). In contrast, many NeuN^+^ cells (80.6 cells/250 μm^2^) and few F-J B^+^ cells (3.4 cells/250 μm^2^) were detected in the SP of the tFI+OXC group on Day 4 after tFI (Figure 8F,f,G,g).

## 3. Discussion

Transient brain ischemia selectively induces neuronal death (DND) in vulnerable structures, such as the neocortex, striatum, and hippocampus [30,31]. The hippocampus is well known to be especially vulnerable to transient ischemia [32,33]. Namely, a massive loss of pyramidal cells (principal neurons) located in the hippocampal CA1 region happens in humans and experimental animals at several days after transient ischemia [34,35]. In this regard, a protective timeline of neuronal death (loss) in ischemic regions has given hope for protective or therapeutic interventions to decrease ischemic injury. However, the underlying mechanisms related to the selectively delayed death of neurons have not yet been fully elucidated. Recently, we reported that pre- and post-treatment with OXC protected against ischemic injury in gerbils [36]. However, the mechanism of neuroprotection is still unclear. Therefore, this study examined the therapeutic effect of OXC treatment after tFI against ischemic injury and its mechanism in a gerbil model of tFI. 

In this study, post-ischemic treatment with OXC significantly reduced the body temperature (to about 33 °C) and significantly attenuated tFI-induced hyperactivity, although therapeutic hypothermia (whole-body cooling) failed to attenuate tFI-induced hyperactivity. Accumulating experimental data have reported that gerbils, after tFI, show hyperactivity in their locomotor activity: the hyperactivity is well addressed on Day 1 after tFI [37,38]. Some studies have reported that ischemia-induced hyperactivity is attenuated by treatments with neuroprotective materials. For instance, in a gerbil model of tFI, pretreatment with fucoidan (a sulfated polysaccharide originating from brown seaweed) attenuates tFI-induced hyperactivity, showing that pyramidal cells of the hippocampal CA1 region are saved from ischemic injury [39]. In addition, it has been reported that pretreatment with rufinamide (a voltage-gated sodium channel blocker) ameliorates tFI-induced hyperactivity in gerbils, showing that CA1 pyramidal neurons are not damaged after tFI [40].

It is well known that the role of the hippocampus in brain function is closely related to learning and memory function [41,42]. Cognitive dysfunction, including decline in short-term memory and spatial memory, can be detected by simple behavioral tests, such as the RAMT (for spatial memory) and PAT (for short-term memory) [43,44]. In our current study, post-ischemic treatment with OXC significantly improved cognitive impairment (decline in spatial and short-term memory) by the RAMT and PAT, respectively, although whole-body cooling failed to improve the tFI-induced cognitive impairment. Previous studies showed that tFI-induced cognitive deficit is improved by neuroprotectants. For instance, administration of Pycnogenol^®^ (a standardized extract of French maritime pine tree bark) before ischemic insults ameliorates decline in short-term memory and spatial memory in a gerbil model of tFI [43]. In addition, treatment with chlorogenic acid (an ester of caffeic acid and quinic acid) significantly moderates tFI-induced cognitive dysfunction in gerbils [44]. These papers show that such cognitive dysfunction is accompanied by delayed neuronal death in the hippocampus. Based on the findings mentioned above, we assessed whether OXC treatment after tFI therapeutically protected neurons in the hippocampus following tFI using CV histochemistry, NeuN immunohistochemistry, and F-J B staining, and we found that post-treatment with OXC effectively protected CA1 pyramidal neurons from ischemic injury induced by tFI. 

It has been reported that hypothermia is one of the most powerful neuroprotective strategies against ischemic injuries [45,46,47]. Moyer et al. (1992) reported that, in a rat model of 60-min transient focal brain ischemia, intra-ischemic hypothermia (about 32 °C immediately induced after the ischemia) decreased infarct volume, whereas delaying cooling did not show a significant effect [48]. In addition, Yamamoto et al. (1999) reported that, in a rat model of traumatic brain injury coupled with hypoxia and hypotension, hypothermia (about 30 °C) reduced supraventricular subcortical neuronal damage [47]. However, there are reports showing that therapeutic hypothermia does not display a considerable neuroprotective effect against brain ischemic injuries. Friedman et al. (2001) reported that post-ischemic hypothermia failed to save hippocampal pyramidal neurons in a rat model of 10-min transient global brain ischemia [49]. In our current experiment, post-ischemic hypothermia did not protect against the death of CA1 pyramidal neurons induced by tFI in gerbils. Generally, it is known that hypothermia is best protective when hypothermia (32 to 35 °C) is induced as soon as possible after ischemia onset and lasts at least one to two hours [50]. Therapeutic hypothermia of human subjects is now obtained in a variety of ways, combining physical cooling of the body surface or blood flow with anesthesia and relaxation to suppress shivering; however, these methods are cumbersome and reveal side effects [9,51]. In this regard, recently, methods using pharmacological agents have been suggested as a way to reduce the core temperature in the body more efficiently and quickly than surface cooling [10]. In our current study, immediate OXC treatment after tFI induced hypothermia within one hour after the reperfusion, and whole-body cooling similar to OXC-induced hypothermia was controlled for six hours after the reperfusion. OXC-induced hypothermia protected CA1 pyramidal cells against death from ischemic injury induced by tFI, but whole-body cooling failed to save the neurons from tFI-induced injury. These findings suggest that there is a critical interaction between body temperature and OXC that appears to permit neuroprotection after ischemic insults, as described below.

Body temperature control is a complex biological process controlled not only by the hypothalamus but also by peripheral thermo-sensors [21]. It has been demonstrated that hypothermia-inducing drugs effectively promote hypothermia in multiple species, including humans [52,53,54]. Ion channels of the transient receptor potential family are pivotal in the regulation of body temperature and engage in the peripheral mechanisms that detect high and low temperature [22]. A central member of the transient receptor potential family is TRPV1, formerly known as capsaicin–vanilloid receptor-1 [55]. TRPV1 receptor is apparently expressed in adult brains, including in the hippocampus, cortex, and hindbrain [23,24], and has an ability to detect variation in body temperature [56]. TRPV1 receptor activation is known to be involved in hypoxic preconditioning in rat hearts [55] and in remote ischemic postconditioning in isolated rat hearts [56], protecting the hearts against ischemia–reperfusion injury. Moreover, Cao et al. [57] demonstrated that pharmacological activation of the TRPV1 channel induces hypothermia and produces neuroprotective effects against ischemia/reperfusion-induced injury in conscious C57BL/6 WT and TRPV1 knockout mice, showing that treatment with TRPV1 agonist (dihydrocapsaicin) at the onset of reperfusion induces hypothermia (33 °C), reduces infarction, and improves neurological function in ischemia-affected mice, but hypothermic and neuroprotective effects were not shown in TRPV1-knockout mice [57]. In addition, capsaicin (a classic TRPV1 channel agonist) produces hypothermia through the activation of centrally located TRPV1-containing neurons [21]. In our current study, OXC (200 mg/kg) treatment after tFI enhanced TRPV1 expression in pyramidal cells located in the hippocampal CA1 region at 30 min after the start of reperfusion, and the increased expression was maintained until four days post-ischemia. Based on our and previous results, OXC produces hypothermia to protect against neuronal death through TRPV1.

TRPV4, a nonselective cation channel, is also expressed in brains [58,59] and acts as an osmotic sensor that mediates changes in osmotic pressure in response to cellular reactions [60]. In addition, TRPV4 activation has been suggested to mediate neuronal and glial responses to swelling in the retina [61]. However, it remains unclear whether TRPV4 is activated via temperature-dependent mechanisms in the process of brain ischemia. Here, we examined the possible involvement of TRPV4 activation in the hippocampus following OXC treatment after tFI using Western blot and immunohistochemistry for TRPV4, and we found that post-treatment with OXC did not significantly increase TRPV4 expression in gerbil hippocampus after tFI. 

In conclusion, the results of our present study show that OXC induced hypothermia through activation of TRPV1 and provided neuroprotection against ischemic injury induced by tFI in gerbils. This study provides evidence showing that thermoreceptor targeting can be an effective strategy for ischemia treatment in conscious subjects, even though the treatment is immediately performed after reperfusion. Therefore, OXC can be introduced to lower the body temperature rapidly and may be applied to patients for hypothermic therapy.

## 4. Materials and Methods

### 4.1. Experimental Animals

Male gerbils (total number = 252) were used at the age of 6 months (body weight, 63–78 g). The gerbils were bred in the Experimental Animal Center of Kangwon National University (Chuncheon, Korea). For this study, the experimental protocol was approved (approval no. KW-200113-1; approval date, 18 February 2020) by the Institutional Animal Care and Use Committee. The research protocol adhered to the guidelines suggested in the “Current International Laws and Policies” of the *Guide for the Care and Use of Laboratory Animals* published by The National Academies Press (8th Ed., 2011).

### 4.2. Experimental Groups, Induction of tFI, and HyT and OXC Treatments

In order to prove the protective effects of OXC against ischemic injury following tFI in gerbils, the gerbils were divided into six groups: (1) sham+vehicle group (*n* = 24); (2) tFI+vehicle group (*n* = 60); (3) sham+HyT group (*n* = 24); (4) tFI+HyT group (*n* = 60); (5) sham+OXC (200 mg/kg) group (*n* = 24); and (6) tFI+OXC group (*n* = 60). Seven and five gerbils in the three tFI groups were used for Western blot analysis and histological examination, respectively, at 30 min, 12 h, 1 day, 2 days, and 4 days after the tFI operation, and in the three sham groups, seven and five gerbils were used at 30 min and 4 days after the sham operation to minimize the numbers. 

In this experiment, tFI was developed as previously described [34]. In short, the gerbils were anesthetized with 2.5% isoflurane (in 32% oxygen and 68% nitrous oxide). Under anesthesia, both common carotid arteries were isolated from the carotid sheath and occluded with aneurysm clips (Yasargil FE 723K) (Aesculap, Tuttlingen, Germany) for five minutes. The perfect stop of blood supply to the brain was confirmed through the observation of arterial blood flow in both retinal arteries (branches of internal carotid arteries) using an ophthalmoscope (HEINE K180^®^) from Heine Optotechnik (Herrsching, Germany). The body temperature before and during the surgery in all groups was controlled at normothermia (37 ± 0.2 °C) using a thermometric blanket. After five minutes of occlusion, the clips were removed. In this study, a sham operation was done by subjecting them to the same tFI surgery without the occlusion of the common carotid arteries.

HyT in the two sham+HyT and tFI+HyT groups was controlled (similar to the change in body temperature in the tFI+OXC group) by whole-body cooling with an ice pack for six hours. The body temperature of the two sham+OXC and tFI+OXC groups was recorded for six hours after immediate intraperitoneal injection of 200 mg/kg OXC (Sigma–Aldrich, St. Louis, MO, USA) after the tFI operation. The dosage of OXC was selected based on a previous study reporting that 200 mg/kg OXC effectively protected against cell death in the brain after tFI [36]. 

To record body temperature change, the body temperature was measured in the rectum every one hour after tFI, over a 6 h period, under ambient room temperature (about 22 °C). The gerbils received a recovery time of four days after tFI because pyramidal cells located in the hippocampal CA1 region begin to die at four days after tFI [30,34,62]. 

### 4.3. SMA Test

The SMA test was performed to examine changes in hyperactivity in all groups. In short, as described previously [63], the SMA test was done on Day 1 after tFI since locomotor activity reaches the highest point on Day 1 after ischemic injury following tFI. The gerbils of all groups received environmental adaptation for two hours, and they were placed onto an open field cage (width, 44 cm; length, 44 cm; height, 30) obtained from Ugo Basile SRL (Gemonio, Italy), in which two parallel horizontal infrared beams 4 × 8 off the floor were installed, for one hour. SMA was recorded using a Photobeam Activity System-Home Cage from San Diego Instruments (San Diego, CA, USA). Movement (trajectory and total distance traveled) was detected through interruption of the array of infrared beams produced by photocells. SMA was continuously monitored for one hour, and the data were collected using an AMB analyzer from IPC Electronics (Cumbria, UK). The data collection was initiated 15 min following habituation in the open field cage. Finally, the obtained results were evaluated as the distance (meters) of movement in the test period (one hour).

### 4.4. Tests of Cognitive Functions

#### 4.4.1. RAMT

To compare spatial memory across all groups, the RAMT was carried out according to precedent studies [38,44]. A radial 8-arm maze from Stoelting Co (Wood Dale, IL, USA) was used for this test. The maze instrument consisted of a central platform and eight arms (each arm width, 5 cm; height, 9 cm; length, 35 cm). The gerbils were trained once a day for three days before tFI. Namely, pellet feed obtained from DBL Co (Chungbuk, Korea) was put at the end part of each arm, and each gerbil was placed onto the central platform. Thereafter, the gerbil looked for the feed. After sham or tFI operation, the real test was carried out once a day for four days beginning one day after the operation. For the analysis, the number of errors was evaluated, with one error occurring every time the gerbil entered an arm that was already visited before. The test was finished when the gerbil consumed the feed.

#### 4.4.2. PAT

To compare short-term memory across the groups, the PAT was conducted according to previously reported methods [64,65] with some modification. In short, the gerbils were tested using the Gemini Avoidance System (GEM 392) from San Diego Instruments (San Diego, CA, USA), which consists of two (dark and light) compartments that communicate each other via a vertically sliding gate. The experimental sessions were performed in two phases: a training session and a real test session performed at one day before and four days after the tFI or sham operation. The real test was performed at 20 min after the training session by measuring the latency time (seconds) during the stay in the dark room. Namely, in the training session, the gerbil was allowed to explore the two compartments freely for one minute while the gate was opened. Thereafter, when the gerbil went into the dark compartment, the door was closed and the gerbil was given an electric foot-shock (0.5 mA) from a steel grid on the floor for five seconds. In the real test session, on Day 4 after tFI, the gerbil was placed onto the light compartment, and the latency time in the light compartment before entering the dark compartment was recorded.

### 4.5. Western Blot Analysis for TRPV1 and TRPV4

To examine the expression levels of TRPV1 and TRPV4 in gerbil hippocampal CA1, the Western blot technique was performed according to previously described methods [66,67]. Briefly, according to the designated time schedule (30 min, 12 h, 1 day, 2 days, and 4 days after sham or tFI operation), gerbils (*n* = 5 for each group) were given anesthesia for euthanasia by intraperitoneal injection with 200 mg/kg pentobarbital sodium (JW pharm. Co., Ltd., Seoul, Korea). Thereafter, their brains were harvested and homogenized with 50 mM phosphate-buffered saline (PBS, pH 7.4) containing 0.1 mM ethylene glycol-bis (β-aminoethyl ether)-N,N,N′,N′-tetraacetic acid (EGTA) (pH 8.0), 10 mM ethylenediaminetetraacetic acid (EDTA) (pH 8.0), 0.2% Nonidet P-40, 15 mM sodium pyrophosphate, 100 mM β-glycerophosphate, 2 mM sodium orthovanadate, 50 mM NaF, 150 mM NaCl, 1 mM phenylmethylsulfonyl fluoride (PMSF), and 1 mM dithiothreitol (DTT). Next, the samples were centrifuged, and the supernatants were taken to determine protein levels using a Micro BCA assay kit from Thermo Fisher Scientific Inc (Waltham, MA, USA) with bovine serum albumin from Pierce Chemical Co (Rockford, IL, USA). Aliquots including 20 μg of total protein were boiled in loading buffer, 150 mM Tris (pH 6.8) containing 6% sodium dodecyl sulfate (SDS), 3 mM DTT, 0.3% bromophenol blue, and 30% glycerol. The samples were separated via 10% SDS-polyacrylamide gel electrophoresis (PAGE). Next, the gels were transferred to nitrocellulose membranes from Pall Co (East Hills, NY, USA) at 350 mA and 4 °C for 90 min. To block non-specific staining, the membranes were incubated in 5% defatted milk for 60 min at room temperature. Thereafter, they were immunoreacted with each primary antibody: rabbit anti-TRPV1 (diluted 1:1000) (Abcam, Cambridge, UK), rabbit anti-TRPV4 (diluted 1:1000) (Abcam), and rabbit anti-β-actin (diluted 1:2000) (Sigma-Aldrich, St. Louis, MO, USA) at 4 °C for 7 h. Subsequently, they were reacted with horseradish peroxidase (HRP)-conjugated donkey anti-rabbit IgG (diluted 1:4500) (Santa Cruz Biotechnology, Santa Cruz, CA, USA) at room temperature for 1 h. Finally, a luminol-based chemiluminescence kit from Thermo Fisher Scientific Inc (Waltham, MA, USA) was used to enhance visualization.

As described previously [68], the immunoblots of TRPV1 and TRPV4 were analyzed using Scion Image software from Scion Crop (Frederick, MD, USA). The bands were scanned, and densitometric analysis was performed. The protein levels were normalized versus the corresponding level of β-actin.

### 4.6. Preparation of Histological Sections

For immunohistochemical and histopathological examinations, gerbils (*n* = 7 for each group) were sacrificed according to the designated time schedule (30 min, 12 h, 1 day, 2 days, and 4 days after tFI or sham operation). As previously described [34], the gerbils were deeply anesthetized with pentobarbital sodium (200 mg/kg) (JW Pharmaceutical, Seoul, Korea). Under the anesthesia, the gerbils were rinsed transcardially with 0.1 M phosphate-buffered saline (pH 7.4) and fixed with 4% paraformaldehyde (in 0.1 M phosphate-buffer, pH 7.4). Subsequently, their brains were obtained and post-fixed using the same fixative for six hours. Thereafter, the brain tissues were cut (25 μm thickness of coronal planes) in a cryostat (Leica, Wetzlar, Germany).

### 4.7. Histochemical Staining Using CV

In order to examine the morphological and neuronal damage in the hippocampus of each group, cresyl violet (CV) staining was performed as we described previously [69]. In brief, cresyl violet acetate (Sigma-Aldrich, St. Louis, MO, USA) was dissolved at 1.0% (*w*/*v*) in distilled water, and glacial acetic acid (0.28%) was added to this solution. The sections were stained and mounted with Canada balsam (Kanto, Tokyo, Japan).

### 4.8. F-J B Staining

F-J B staining was performed to examine neuronal degeneration (death or loss). According to published procedure [34], the prepared brain sections were soaked in 1% sodium hydroxide, immediately transferred to 0.06% potassium permanganate, and promptly reacted with 0.0004% Fluoro-Jade B (Histochem, Jefferson, AR, USA). The sections were briefly washed and put on a slide warmer (about 50 °C) for reaction with F-J B. To appraise the therapeutic effect of OXC against tFI, the numbers of F-J B^+^ cells were counted in the CA1 region according to a method published in [70]. Briefly, digital images of F-J B^+^ cells were captured from five sections per gerbil using an epifluorescent microscope (Carl Zeiss) (Oberkochen, Germany) at 450–490 nm wavelength. The cells were counted in 250 μm^2^, which included the SP, at the center of the CA1 region using an image analyzing system (Optimas 6.5) (CyberMetrics, Scottsdale, AZ, USA).

### 4.9. Immunohistochemistry

In order to investigate changes in NeuN, TRPV1, and TRPV4 immunoreactivity in the CA1 region, general immunohistochemistry was performed. In brief, according to a published method [34], the prepared brain sections were incubated with primary antibodies—mouse anti-NeuN (diluted, 1:1100) (Chemicon, Temecula, CA, USA), mouse anti-TRPV1 (diluted, 1:500) (Abcam, Cambridge, UK), and rabbit anti-TRPV4 (diluted, 1:500) (Abcam, Cambridge, UK). Thereafter, these incubated sections were incubated in the corresponding secondary antibodies (diluted, 1:250) (Vector Laboratories Inc., Burlingame, CA, USA) and developed using Vectastain ABC (diluted, 1:250) (Vector Laboratories Inc., Burlingame, CA, USA). Finally, these immunoreacted sections had color after visualization with 3,3’-diaminobenzidine. The numbers of NeuN^+^ cells were counted as follows. Digital images of NeuN^+^ cells were captured from five sections per gerbil using a light microscope (AxioM1) (Carl Zeiss, Germany). The cells were counted in the same way as the F-J B^+^ cell count. To evaluate the density of TRPV1^+^ and TRPV4^+^ structures, the corresponding areas in the CA1 region were used in five sections per animal. Images of the TRPV1^+^ and TRPV4^+^ structures were captured using an AxioM1 light microscope (Carl Zeiss) (Germany). The densities of TRPV1^+^ and TRPV4^+^ structures were evaluated as the relative optical density (ROD). To this end, the images were transformed to the mean gray level. The ROD was presented as a percentage using Adobe Photoshop (version 8.0) and NIH Image J software (National Institutes of Health, Bethesda, MD, USA).

### 4.10. Statistical Analysis

We presented the data as the means ± standard error of the mean (SEM). All statistical analyses were performed with the aid of GraphPad Prism (version 5.0) (GraphPad Software, La Jolla, CA, USA). Differences in the means among the experimental groups were statistically analyzed by two-way analysis of variance (ANOVA) with a post hoc Bonferroni’s multiple comparison test to elucidate tFI-related differences among all groups. Statistical significance was considered at *p* < 0.05.

## Data Availability

The data presented in this study are available on request from the corresponding author.

## Abbreviations

CA1: subfield Cornu Ammonis 1; CNS, central nervous system; CV, cresyl violet; DG, dentate gyrus; F-J B, Fluoro-Jade B; HyT, hypothermia; NeuN, neuronal nuclei; OXC, oxcarbazepine; PAT, passive avoidance test; ROD, relative optical density; SMA, spontaneous motor activity; SO, stratum oriens; SP, stratum pyramidale; SR, stratum radiatum; tFI, transient forebrain ischemia; TRPV1, transient receptor potential vanilloid type 1.
